# 1-Cyano­methyl-1,4-diazo­niabicyclo­[2.2.2]octane tetra­chloridocuprate(II)

**DOI:** 10.1107/S1600536810047501

**Published:** 2010-11-24

**Authors:** Bin Wei

**Affiliations:** a Ordered Matter Science Research Center, Southeast University, Nanjing 211189, People’s Republic of China

## Abstract

In the crystal structure of the title compound, (C_8_H_15_N_3_)[CuCl_4_], the cations and anions, in which the Cu^II^ atom is tetra­hedrally coordinated, are linked *via* N—H⋯Cl hydrogen bonds into chains that are elongated in the *c*-axis direction.

## Related literature

For a similar structure, see: Wen *et al.* (2004 ▶). For our ongoing investigations of DABCO derivatives, see: Chen *et al.* (2010 ▶); Zhang *et al.* (2009 ▶).
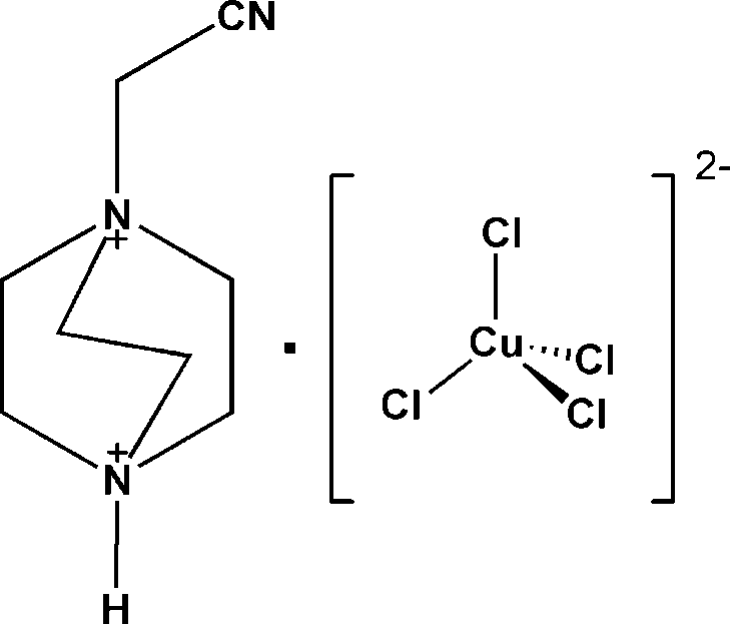


## Experimental

### 

#### Crystal data

(C_8_H_15_N_3_)[CuCl_4_]
*M* *_r_* = 358.57
Monoclinic, 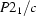

*a* = 8.2714 (6) Å
*b* = 13.6585 (8) Å
*c* = 12.1636 (10) Å
β = 96.501 (5)°
*V* = 1365.35 (17) Å^3^
*Z* = 4
Mo *K*α radiation
μ = 2.36 mm^−1^
*T* = 293 K
0.2 × 0.2 × 0.2 mm

#### Data collection

Rigaku Mercury CCD diffractometer
Absorption correction: multi-scan (*CrystalClear*; Rigaku, 2005 ▶) *T*_min_ = 0.641, *T*_max_ = 1.000
14635 measured reflections
3123 independent reflections
2307 reflections with *I* > 2σ(*I*)
*R*_int_ = 0.055

#### Refinement

*R*[*F* ^2^ > 2σ(*F* ^2^)] = 0.042
*wR*(*F* ^2^) = 0.118
*S* = 1.09
3123 reflections
145 parameters
H-atom parameters constrained
Δρ_max_ = 1.00 e Å^−3^
Δρ_min_ = −1.00 e Å^−3^

### 

Data collection: *CrystalClear* (Rigaku, 2005 ▶); cell refinement: *CrystalClear*; data reduction: *CrystalClear*; program(s) used to solve structure: *SHELXS97* (Sheldrick, 2008 ▶); program(s) used to refine structure: *SHELXL97* (Sheldrick, 2008 ▶); molecular graphics: *DIAMOND* (Brandenburg & Putz, 2005 ▶); software used to prepare material for publication: *SHELXL97*.

## Supplementary Material

Crystal structure: contains datablocks I, global. DOI: 10.1107/S1600536810047501/nc2197sup1.cif

Structure factors: contains datablocks I. DOI: 10.1107/S1600536810047501/nc2197Isup2.hkl

Additional supplementary materials:  crystallographic information; 3D view; checkCIF report

## Figures and Tables

**Table 1:** Hydrogen-bond geometry (Å, °)

*D*—H⋯*A*	*D*—H	H⋯*A*	*D*⋯*A*	*D*—H⋯*A*
N1—H1*C*⋯Cl3^i^	0.94	2.58	3.325 (3)	136
N1—H1*C*⋯Cl1	0.94	2.70	3.247 (3)	118
N1—H1*C*⋯Cl2	0.94	2.80	3.441 (3)	126

Symmetry code: (i) 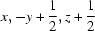.

**Fig. 1.**
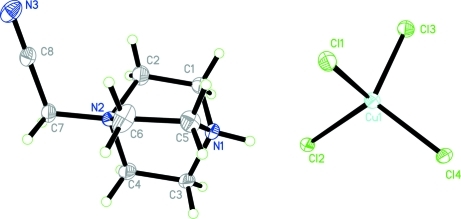
The asymmetric unit of compound (I) with labelling and displacement ellipsoids drawn at the 30% probability level.

**Fig. 2.**
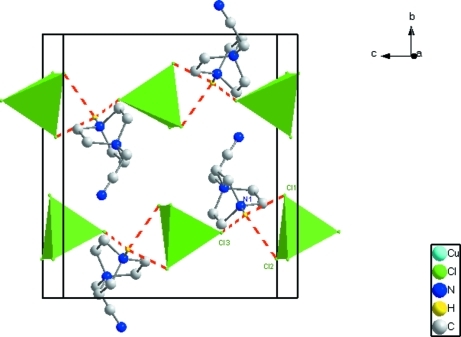
Crystal structure of the title compound with view along the a axis. C—H H atoms have been omitted for clarity. Intermolecular N—-H···Cl interactions are shown as dashed lines.

## supplementary crystallographic information

### Comment

Only a few crystal structures containing the 1-(cyanomethyl)-4-aza-1-azonia-bicyclo-[2.2.2]octane [C_8_H_15_N_3_[^+^ cation have been determined. In our ongoing investigations in the field of DABCO derivative some of which may be ferroelectrics (Zhang *et al.*, 2009; Chen *et al.* 2010) the title compound was prepared and characterized by single-crystal X-ray diffraction.

The asymmetric unit of the title compound contains one [C_8_H_15_N_3_[^+^ cation and one [CuCl_4_]^2-^ anion in general positions (Fig. 1). The Cu atoms are coordinated by four Cl atoms with distances in the range of 2.246 (2) to 2.308 (6) Å. The Cl—Cu—Cl bond angles are between 99.01 (5) and 126.10 (5)° which shows that the coordination polyhedron can be described as a strongly distorted tetrahedron. The structure of the [CuCl_4_]^2-^ anion is close to those observed in similar complexes, like in (C_10_H_10_N2S)[CuCl_4_] (Wen *et al.*, 2004). The organic cations and the complex anions are linked by N—H···Cl hydrogen-bonding interactions (Fig. 2 and Tab. 1). The N—H H atom attached to N1 interacts two Cl atoms of one [CuCl_4_]^2-^ anion and one Cl atom of a further adjacent [CuCl_4_]^2-^ anion with N1—H1C···Cl1, N1—H1C···Cl2 and N1—H1C···Cl3^i^ distances of 2.70, 2.80 and 2.58Å, respectively [symmetry codes: (i) *x*, -*y* + 1/2, *z* + 1/2].

### Experimental

Bromoacetonitrile (0.1 mol, 12.00 g) was added to a CH_3_CN (25 ml) solution of 1,4-Diaza-bicyclo[2.2.2]octane (DABCO) (0.05 mol, 5.6 g) with stirring for 1 h at room temperature. 1-(cyanomethyl)-4-aza-1-azonia-bicyclo[2.2.2]octane bromide quickly formed as white solid was filtered, washed with acetonitrile and dried (yield: 80%). CuCl_2_.2H_2_O (0.001 mol, 0.171 g) and 2 ml 36% HCl were dissolved in MeOH (20 ml) and 1-(cyanomethyl)-4-aza-1-azonia-bicyclo[2.2.2]octane bromide (0.002 mol, 0.464 g) in H_2_O (20 ml) was added. The resulting solution was stirred until a clear solution was obtained. After slow evaporation of the solvent, red-brown block crystals of the title compound suitable for X-ray analysis were obtained in about 60% yield.

### Refinement

The C—H H atoms were positioned with idealized geometry and refined using a riding model (*U*_iso_(H) = 1.2 *U*_eq_(C).

### Crystal data

**Table:**

(C_8_H_15_N_3_)[CuCl_4_]	*F*(000) = 724
*M**_r_* = 358.57	*D*_x_ = 1.744 Mg m^−^^3^
Monoclinic, *P*2_1_/*c*	Mo *K*α radiation, λ = 0.71073 Å
Hall symbol: -P 2ybc	Cell parameters from 3825 reflections
*a* = 8.2714 (6) Å	θ = 2.5–27.5°
*b* = 13.6585 (8) Å	µ = 2.36 mm^−^^1^
*c* = 12.1636 (10) Å	*T* = 293 K
β = 96.501 (5)°	Block, red-brown
*V* = 1365.35 (17) Å^3^	0.2 × 0.2 × 0.2 mm
*Z* = 4	

### Data collection

**Table:**

Rigaku Mercury CCD diffractometer	3123 independent reflections
Radiation source: fine-focus sealed tube	2307 reflections with *I* > 2σ(*I*)
graphite	*R*_int_ = 0.055
Detector resolution: 28.5714 pixels mm^-1^	θ_max_ = 27.5°, θ_min_ = 2.5°
ω scans	*h* = −10→10
Absorption correction: multi-scan (*CrystalClear*; Rigaku, 2005)	*k* = −17→17
*T*_min_ = 0.641, *T*_max_ = 1.000	*l* = −15→15
14635 measured reflections	

### Refinement

**Table:**

Refinement on *F*^2^	Secondary atom site location: difference Fourier map
Least-squares matrix: full	Hydrogen site location: inferred from neighbouring sites
*R*[*F*^2^ > 2σ(*F*^2^)] = 0.042	H-atom parameters constrained
*wR*(*F*^2^) = 0.118	*w* = 1/[σ^2^(*F*_o_^2^) + (0.0585*P*)^2^] where *P* = (*F*_o_^2^ + 2*F*_c_^2^)/3
*S* = 1.09	(Δ/σ)_max_ < 0.001
3123 reflections	Δρ_max_ = 1.00 e Å^−^^3^
145 parameters	Δρ_min_ = −1.00 e Å^−^^3^
0 restraints	Extinction correction: *SHELXL97* (Sheldrick, 2008)
Primary atom site location: structure-invariant direct methods	Extinction coefficient: 0.0055 (11)

### Special details

**Table:**

Geometry. All e.s.d.'s (except the e.s.d. in the dihedral angle between two l.s. planes) are estimated using the full covariance matrix. The cell e.s.d.'s are taken into account individually in the estimation of e.s.d.'s in distances, angles and torsion angles; correlations between e.s.d.'s in cell parameters are only used when they are defined by crystal symmetry. An approximate (isotropic) treatment of cell e.s.d.'s is used for estimating e.s.d.'s involving l.s. planes.
Refinement. Refinement of *F*^2^ against ALL reflections. The weighted *R*-factor *wR* and goodness of fit *S* are based on *F*^2^, conventional *R*-factors *R* are based on *F*, with *F* set to zero for negative *F*^2^. The threshold expression of *F*^2^ > σ(*F*^2^) is used only for calculating *R*-factors(gt) *etc*. and is not relevant to the choice of reflections for refinement. *R*-factors based on *F*^2^ are statistically about twice as large as those based on *F*, and *R*- factors based on ALL data will be even larger.

### Fractional atomic coordinates and isotropic or equivalent isotropic displacement parameters (Å^2^)

**Table:**

	*x*	*y*	*z*	*U*_iso_*/*U*_eq_	
Cu1	0.72905 (6)	0.23247 (4)	−0.01548 (4)	0.02929 (17)	
Cl2	0.51578 (11)	0.14877 (7)	0.04825 (7)	0.0260 (2)	
Cl3	0.74393 (12)	0.25165 (8)	−0.19747 (8)	0.0354 (3)	
Cl4	0.95764 (12)	0.14720 (8)	0.03958 (8)	0.0352 (3)	
Cl1	0.67871 (13)	0.38977 (7)	0.02896 (9)	0.0358 (3)	
N1	0.3987 (4)	0.3497 (2)	0.1903 (3)	0.0289 (7)	
H1C	0.4947	0.3162	0.1788	0.035*	
C8	−0.0796 (5)	0.5439 (3)	0.2033 (3)	0.0309 (9)	
C3	0.3247 (5)	0.2807 (3)	0.2658 (3)	0.0314 (9)	
H3A	0.3944	0.2750	0.3352	0.038*	
H3B	0.3131	0.2163	0.2322	0.038*	
N2	0.1337 (3)	0.4183 (2)	0.2368 (2)	0.0230 (7)	
C1	0.2879 (5)	0.3613 (3)	0.0877 (3)	0.0338 (9)	
H1A	0.2638	0.2978	0.0543	0.041*	
H1B	0.3394	0.4012	0.0356	0.041*	
C7	−0.0253 (5)	0.4565 (3)	0.2655 (3)	0.0321 (9)	
H7A	−0.1071	0.4058	0.2514	0.039*	
H7B	−0.0153	0.4715	0.3440	0.039*	
C5	0.4289 (5)	0.4464 (3)	0.2451 (4)	0.0372 (10)	
H5A	0.4701	0.4923	0.1940	0.045*	
H5B	0.5097	0.4397	0.3089	0.045*	
N3	−0.1269 (5)	0.6094 (3)	0.1516 (3)	0.0440 (10)	
C2	0.1332 (5)	0.4093 (4)	0.1134 (3)	0.0405 (11)	
H2A	0.0405	0.3705	0.0830	0.049*	
H2B	0.1237	0.4737	0.0798	0.049*	
C6	0.2706 (5)	0.4844 (3)	0.2810 (4)	0.0479 (12)	
H6A	0.2783	0.4865	0.3611	0.057*	
H6B	0.2501	0.5503	0.2531	0.057*	
C4	0.1615 (5)	0.3192 (3)	0.2863 (4)	0.0451 (12)	
H4A	0.0774	0.2749	0.2544	0.054*	
H4B	0.1552	0.3226	0.3654	0.054*	

### Atomic displacement parameters (Å^2^)

**Table:**

	*U*^11^	*U*^22^	*U*^33^	*U*^12^	*U*^13^	*U*^23^
Cu1	0.0302 (3)	0.0303 (3)	0.0276 (3)	0.0024 (2)	0.0041 (2)	0.0008 (2)
Cl2	0.0255 (4)	0.0261 (4)	0.0266 (5)	0.0000 (4)	0.0044 (4)	0.0034 (4)
Cl3	0.0337 (5)	0.0479 (6)	0.0246 (5)	0.0038 (4)	0.0025 (4)	0.0020 (4)
Cl4	0.0341 (5)	0.0433 (6)	0.0286 (5)	0.0146 (4)	0.0050 (4)	0.0086 (4)
Cl1	0.0400 (6)	0.0261 (5)	0.0434 (6)	−0.0009 (4)	0.0143 (5)	−0.0009 (4)
N1	0.0214 (16)	0.0337 (18)	0.0318 (18)	0.0053 (14)	0.0048 (14)	0.0047 (15)
C8	0.029 (2)	0.030 (2)	0.033 (2)	0.0022 (17)	0.0030 (17)	−0.0057 (18)
C3	0.027 (2)	0.033 (2)	0.035 (2)	0.0029 (17)	0.0051 (17)	0.0107 (18)
N2	0.0223 (15)	0.0210 (15)	0.0255 (17)	−0.0001 (12)	0.0015 (12)	0.0021 (13)
C1	0.034 (2)	0.044 (3)	0.023 (2)	0.0066 (18)	0.0025 (17)	0.0013 (18)
C7	0.030 (2)	0.032 (2)	0.036 (2)	0.0047 (17)	0.0113 (17)	−0.0007 (18)
C5	0.030 (2)	0.038 (2)	0.043 (3)	−0.0091 (18)	0.0040 (19)	−0.002 (2)
N3	0.052 (2)	0.032 (2)	0.045 (2)	0.0094 (17)	−0.0071 (19)	−0.0073 (18)
C2	0.037 (2)	0.059 (3)	0.025 (2)	0.011 (2)	0.0003 (18)	−0.004 (2)
C6	0.034 (2)	0.038 (3)	0.068 (3)	−0.0033 (19)	−0.010 (2)	−0.017 (2)
C4	0.046 (3)	0.034 (2)	0.059 (3)	0.013 (2)	0.024 (2)	0.024 (2)

### Geometric parameters (Å, °)

**Table:**

Cu1—Cl3	2.2463 (11)	N2—C2	1.506 (5)
Cu1—Cl4	2.2568 (10)	C1—C2	1.502 (5)
Cu1—Cl1	2.2655 (11)	C1—H1A	0.9700
Cu1—Cl2	2.3085 (10)	C1—H1B	0.9700
N1—C1	1.471 (5)	C7—H7A	0.9700
N1—C5	1.488 (5)	C7—H7B	0.9700
N1—C3	1.495 (5)	C5—C6	1.518 (6)
N1—H1C	0.9405	C5—H5A	0.9700
C8—N3	1.137 (5)	C5—H5B	0.9700
C8—C7	1.458 (6)	C2—H2A	0.9700
C3—C4	1.496 (5)	C2—H2B	0.9700
C3—H3A	0.9700	C6—H6A	0.9700
C3—H3B	0.9700	C6—H6B	0.9700
N2—C4	1.488 (5)	C4—H4A	0.9700
N2—C7	1.492 (4)	C4—H4B	0.9700
N2—C6	1.499 (5)		
			
Cl3—Cu1—Cl4	102.42 (4)	C8—C7—N2	113.1 (3)
Cl3—Cu1—Cl1	99.01 (4)	C8—C7—H7A	109.0
Cl4—Cu1—Cl1	126.09 (5)	N2—C7—H7A	109.0
Cl3—Cu1—Cl2	121.19 (4)	C8—C7—H7B	109.0
Cl4—Cu1—Cl2	106.93 (4)	N2—C7—H7B	109.0
Cl1—Cu1—Cl2	102.79 (4)	H7A—C7—H7B	107.8
C1—N1—C5	110.0 (3)	N1—C5—C6	109.0 (3)
C1—N1—C3	109.3 (3)	N1—C5—H5A	109.9
C5—N1—C3	110.2 (3)	C6—C5—H5A	109.9
C1—N1—H1C	112.4	N1—C5—H5B	109.9
C5—N1—H1C	113.4	C6—C5—H5B	109.9
C3—N1—H1C	101.2	H5A—C5—H5B	108.3
N3—C8—C7	176.8 (4)	C1—C2—N2	109.7 (3)
N1—C3—C4	108.7 (3)	C1—C2—H2A	109.7
N1—C3—H3A	110.0	N2—C2—H2A	109.7
C4—C3—H3A	110.0	C1—C2—H2B	109.7
N1—C3—H3B	110.0	N2—C2—H2B	109.7
C4—C3—H3B	110.0	H2A—C2—H2B	108.2
H3A—C3—H3B	108.3	N2—C6—C5	109.4 (3)
C4—N2—C7	108.9 (3)	N2—C6—H6A	109.8
C4—N2—C6	109.1 (3)	C5—C6—H6A	109.8
C7—N2—C6	110.8 (3)	N2—C6—H6B	109.8
C4—N2—C2	108.2 (3)	C5—C6—H6B	109.8
C7—N2—C2	111.0 (3)	H6A—C6—H6B	108.2
C6—N2—C2	108.8 (3)	N2—C4—C3	110.7 (3)
N1—C1—C2	109.5 (3)	N2—C4—H4A	109.5
N1—C1—H1A	109.8	C3—C4—H4A	109.5
C2—C1—H1A	109.8	N2—C4—H4B	109.5
N1—C1—H1B	109.8	C3—C4—H4B	109.5
C2—C1—H1B	109.8	H4A—C4—H4B	108.1
H1A—C1—H1B	108.2		

### Hydrogen-bond geometry (Å, °)

**Table:**

*D*—H···*A*	*D*—H	H···*A*	*D*···*A*	*D*—H···*A*
N1—H1C···Cl3^i^	0.94	2.58	3.325 (3)	136
N1—H1C···Cl1	0.94	2.70	3.247 (3)	118
N1—H1C···Cl2	0.94	2.80	3.441 (3)	126

Symmetry codes: (i) *x*, −*y*+1/2, *z*+1/2.
